# Toluene exposure and changes in platelet count: a narrative review

**DOI:** 10.47626/1679-4435-2021-896

**Published:** 2024-02-16

**Authors:** Gabriel Machado Romão da Silva, Eric Slawka, Aline de Souza Espíndola Santos, Angelica dos Santos Vianna

**Affiliations:** 1 Faculdade de Medicina, Universidade Federal do Rio de Janeiro (UFRJ), Rio de Janeiro, RJ, Brazil; 2 Instituto de Estudos em Saúde Coletiva, UFRJ, Rio de Janeiro, RJ, Brazil

**Keywords:** toluene, platelet count, humans, review, thrombocytopenia, tolueno, contagem de plaquetas, humanos, revisão, trombocitopenia

## Abstract

Toluene is a widely used solvent whose many toxic effects include neurological and hematological damage. This study reviewed evidence about the effects of toluene exposure on platelet count in humans. Three electronic databases and a digital library of theses and dissertations were searched using a specific strategy, yielding 64 articles, of which 14 were selected. These studies assessed a total of 15,759 participants, including 13,297 exposed individuals, mainly women exposed in an environmental setting. The major findings were: (1) conflicting results (positive, inverse, or no association), (2) cross-contamination with other substances, which impaired assessment of the relationship, and (3) a lack of studies. Thus, further research is needed on this topic, especially toluene exposure in isolation from associated substances.

## Abstract (pt)

O tolueno é um solvente amplamente utilizado com múltiplos efeitos tóxicos, sobretudo sobre o sistema nervoso central, assim como efeitos hematológicos. Este estudo foi conduzido para revisar a evidência presente na literatura sobre a exposição humana ao tolueno e seu efeito na contagem de plaquetas. Em 3 bases de dados eletrônicas e 1 biblioteca digital de teses e dissertações foram pesquisadas utilizando uma estratégia de busca específica, da qual resultaram 64 artigos, dos quais 14 foram selecionados. Estes avaliaram 15.759 pessoas, com 13.297 indivíduos expostos, compostos principalmente de mulheres em um cenário ambiental. Foram encontrados 3 grandes resultados, os quais incluem a presença de relações conflitantes (positiva, inversa, sem associação), a presença frequente de outras substâncias afetando a análise da relação, e a falta de estudos. Portanto, nós recomendamos mais pesquisas no tópico, com ênfase na exposição ao tolueno sem substâncias associadas.

## INTRODUCTION

Solvents are widely used chemical substances that dissolve solutes. Despite various chemical compositions, they share similar properties such as lipophilia, volatility and flammability.^1,2,3^

Toluene is in a group of solvents called aromatic hydrocarbons.^1^ It can be found naturally or can be artificially synthesized. In nature, it occurs in crude oil and balsam of Tolu, a South American tree.^4^ It is used in the processing of fossil fuels and the production of cleaning products, glues, paints, and cosmetics.^1^ It is also among the most widely abused inhaled recreational drugs,^5,6^ despite knowledge of its many toxic effects.

Inhalation is the primary route of exposure, and the compound is rapidly absorbed by the lungs. It is then distributed through the bloodstream, preferentially to fat, brain, bone marrow, liver, and kidney tissue.^4^ Significant amounts can also be absorbed through ingestion and dermal contact, although at a slower rate.^4,5^ Toluene is metabolized into benzoic acid in the liver and, after conjugation with glycine, forms hippuric acid, which is excreted through urine, the main route of elimination.

Toluene has many toxic effects, although the mechanism by which it produces systemic toxicity has not been yet established.^7^ The central nervous system is the primary target of acute and chronic exposure.^6^ It also has toxic effects on the respiratory system (eg, chemical pneumonitis), liver (eg, hepatitis), and kidneys (eg, tubular necrosis).^1^ All of these effects depend on the concentration, length of exposure, and individual susceptibility.^8^

Hematological effects have been associated with toluene exposure, although there has been controversy about cross-contamination with other substances, especially benzene, but also ethylbenzene, and xylene, which, with toluene, form a group abbreviated as BTEX.^9,10,11,12,13,14,15^ Immune thrombocytopenic purpura (ITP), a rare hematological disorder characterized by thrombocytopenia (reduced platelet count [PC]), has also been reported after toluene exposure.^10,11^

This study aims to review evidence in the literature about human toluene exposure and PC changes.

## METHODS

This narrative review collected data about the effects of toluene on PC. In January 2021, we developed a search strategy for 3 electronic databases (BVS/LILACS; Embase and MEDLINE/PubMed) and the Fiocruz ARCA Digital Library of Theses and Dissertations using the following search terms: toluene, thrombocytopenia, thrombocytopenic, platelet, count, hematologic, parameter and measure. To these were added the Boolean operators ("AND" "OR" and "NOT"), and MeSH and DeCS equivalents. The reference lists of the selected articles were also manually reviewed. Articles already indexed by MEDLINE were excluded from the search in BVS/LILACS and Embase to avoid duplicates.

The selection criteria were observational studies, clinical trials, systematic reviews, dissertations, and theses on human exposure to toluene that also included PC, thrombocytopenia, or thrombocytopenic purpura. Only studies published between 1950 and 2021 in English, Spanish, or Portuguese were eligible. We did not include animal or *in vitro* studies, editorials, expert opinions, narrative reviews, or book chapters. We divided the participants into 3 age groups: children and teenagers (aged ≤17 years), adults (aged 18-64 years), and older adults (aged ≥60 years).^16,17^

## RESULTS

**Figure 1:**
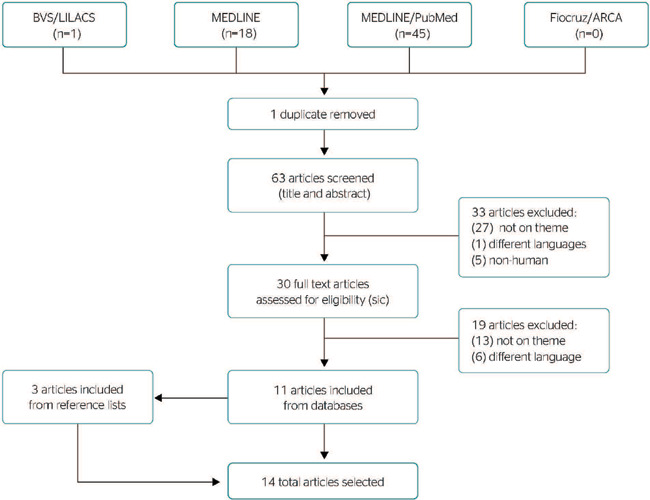
Study selection flowchart.

The main search was performed on January 28, 2021, yielding 64 records, 1 from BVS/LILACS, 18 from Embase, 45 from MEDLINE, and 0 from Fiocruz/ ARCA. After 1 duplicate was removed, 30 records were selected based on title and abstract screening. After full-text analysis, 11 were selected. Three additional articles were included from a reference list search, for a total of 14 articles. The 51 exclusions were due to unrelated themes (40), publication in other languages (7), or animal studies (5). Figure 1 shows the study selection flowchart.

All included studies were observational: 7 were cross-sectional, 4 were case reports, and 3 were cohort studies. They were published between 1963 and 2020, with the majority (85%) published in the last decade. The articles originated from 9 countries: 4 from the United States, 2 each from Nigeria and South Korea, and 1 each from Canada, China, Iran, Mexico, Taiwan, and the United Kingdom.

The studies included a total of 15,759 individuals: 8101 (51.4%) men, 7469 (47.4%) women, and 189 (1.2%) of unknown sex. There were 3041 (19.3%) smokers, 12,163 (77.2%) non-smokers, and 555 (3.5%) with unreported smoking status. Race was reported in 3 studies.^10,12,14^ Of these 9451 (60%) participants, 4129 (43.7%) were White, 2086 (22.1%) were Black, and 3236 (34.2%) were Hispanic or other. No race was reported for the remaining 6308 (40%) participants.

Eight studies (14,962 participants) assessed exposure through biomarker or bioindicator measurement.^9,12,14,15,16,17,18,19,20,21,22^ Biomarker levels were measured by urine sample in 34 (0.2%) participants,^18^ exhaled breath in 97 (0.6%),^19^ and blood sample in 14,279 (90.6%).^9,12,14,22^ Environmental assessment was performed by measuring water and soil samples for 333 (2.1%) participants^20^ and air samples for 219 (1.4%) participants.^21^ Biomarkers levels were not measured for the remaining 797 (5%) participants.

A total of 13,297 (84.4%) participants were considered to have been exposed to toluene, of whom 3824 (28.8%) were men and 4148 (31.2%) were women; 5325 (40%) were not differentiated according to exposure. Of the exposed participants, 1644 (12.4%) were smokers, 6328 (47.6%) were non-smokers, and the remaining 5325 (40%) were not differentiated according to smoking status. No studies specified the race of exposed participants. The remaining 2462 (16.6%) participants were not exposed.

All 14 studies evaluated adults: 9 included adults only (852), 3 also included older adults (1455), and 2 also included children and older adults (13,452). The 5 environmental exposure studies contained more participants (12,096) than the 9 occupational exposure studies (1201 participants).

Among the 13,297 exposed participants, chronic exposure (> 1 month) was more frequent than acute exposure (< 1 month): 12,592 participants (94.7%, 10 studies) vs. 705 participants (5.3%, 4 studies), respectively. The route of exposure was described in 4 studies, comprising 5 participants: respiratory (3 studies, 4 exposed participants),^11,23,24^ and cutaneous (1 study, lexposed participant).^10^

Most studies^9,12,14,19,20,21,22,23,24,25,26^ reported some degree of concomitant exposure to other chemicals, such as aromatic hydrocarbons, carbon tetrachloride, heavy metals, hydrogen chloride, or phosgene. The most common cross-contaminant was benzene, reported in 10 studies.

Ten studies reported a statistical analysis of toluene exposure and PC: 7 used mean difference (Student's *t*-test)^9,18,20,21,22,25,26^, 3 used linear regression (β coefficient)^9,12,14^, 1 used logistic regression (odds ratio [OR])^19^, 1 (data not shown) used the Pearson correlation,^20^ and 1 used prevalence.^19^ The mean difference results were: no difference (4 studies, p > 0.05),^20,21,25,26^ a mean decrease (2 studies, p < 0.01),^9,18^ and a mean increase (1 study, p < 0.05).^22^ The regression coefficient results were no association (2 studies, p > 0.05),^9,14^ an inverse association (1 study β = -18.66, p = 0.01),^12^ and a positive association (1 study, β = 0.07, p = 0.0017).^14^ The OR results were a positive association (1 study, OR = 0.5, 95%CI 0.1-3.0).^19^ The correlation coefficient results were a negative correlation between exposure period and PC (1 study, p < 0.01).^20^ The prevalence results were 7.2% for thrombocytopenia (1 study).^19^

Two^10,26^ the 14 studies specifically reported ITP, although neither could identify an associated substance. One of these studies^10^ described exacerbation of preexisting ITP after continuous toluene exposure, and the other^26^ reported specific exposure to toluene diisocyanate. The included studies are described in Table 1.

**Table 1:** Selected data from the included studies

Author (year/ country)	Study characteristics (design, sample, number of exposed)	Population (sex, age groups, race, smokers)	Exposure characteristics (type, means, period, associated substances)	Biomarker or bioindicator	Statistical analysis
Jennings & Cower^11^ (1963, UK)	Case report; n = 2; ExG = 2	Male = 2; A; N/l; smokers =1	Occupational; respiratory; acute; none	None	None
Doclson^10^ (1966, USA)	Case report; n = 1; ExG = 1	Male = 1; A; White = 1; smokers = 0	Occupational; cutaneous; acute; none	None	None
Shin et al.^18^ (2011, Taiwan)	Cross-sectional; n =34; ExG = 34	Male = 34; A; N/l; smokers = 21	Occupational; N/l; chronic; none	Hippuric acid (urine sample)	Median difference: 216 (CET) X 252 GET) (p = 0.018)
Tharr & Kudla^24^ (1997, USA)	Case report; n = 2; ExG = 2	N/I; A; N/I; N/I	Occupational; N/I; chronic; benzene	None	None
Haro-García et al.^19^ (2012, Mexico)	Cross-sectional; n = 97; ExG = 97	N/I; A; N/I; smokers = 74	Occupational; N/I; chronic; BTEX	BTEX (exhaled breath)	Prevalence of thrombocytopenia: 7.2%; logistic regression: OR = 0.5 (95%CI 0.1-3.0)
Park et al.^23^ (2012, South Korea)	Case-report; n = 1; ExG = 1	Male = 1; A; N/I; N/I	Occupational; respiratory; acute; hydrogen chloride, phosgene, carbon tetrachloride	None	None
Ezejiofor et al.^20^ (2016, Nigeria)	Cohort; n = 333, ExG = 303	Male = 273, female = 60; A, OA; N/I; N/I	Occupational; N/I; chronic; BTEX	BTEX (water and soil samples)	Mean diference: none (p = 0.57); Pearson’s correlation for exposure period and platelets: data not shown (p < 0.01)
Choi et al.^25^ (2017, South Korea)	Cohort; n = 701; ExG = 701	Male = 260, female = 441; A, OA; N/I; smokers = 65	Occupational; N/I; acute; BTEX, polycystic aromatic hydrocarbons, heavy metals	No BTEXS	Mean diference: none (p = 0.532)
Doherty et al.^12^ (2017, USA)	Cross-sectional; n = 406; ExG = 144	Male = 306, female = 100; A; White = 122, Black = 110, other = 15; N/I = 159; smokers = 247	Environmental; N/I; chronic; BTEXS	BTEXS and 2,5-dimethylfuran (blood sample)	Linear regression: β for non-smokers = -18.66 (p = 0.01)
Chen et al.^9^ (2019, China)	Cross-sectional; n = 421; ExG = 240	Male = 210, female = 211; N/I; smokers = 131	Environmental; N/I; chronic; BTEX	BTEX (blood sample)	Mean diference: ExG (178) vs CG (192) (p < 0.01); linear regression: none
Eze et al.^26^ (2019, Nigeria)	Cohort; n = 90; ExG = 60	N/I; A; N/I; smokers = 0	Occupational; N/I; chronic; BTEX	None	Mean diference: none (p > 0.05)
Samadi et al.^21^ (2019, Iran)	Cross-sectional; n = 219; ExG = 148	Male = 212, female = 7; N/I; N/I	Environmental; N/I; chronic; BTEXS	BTXS (air sample)	Median diference: none (p = 0.629)
Watson et al.^14^ (2021, USA)	Cross-sectional; n = 9502; ExG = 7732	Male = 4906, female = 4596; White = 4006, Black = 1976, Hispanic = 1964, other = 1257; smokers = 3512	Environmental; N/I; chronic; BTEXS	BTEXS (blood sample)	Linear regression for non-smokers: β = 0.070 (95%CI 2.281-9.177, p = 0.0017); linear regression for smokers: β = 0.040 (95%CI -3.421-10.192, p = 0.3218)
Cakmak et al.^22^ (2020, Canada)	Cross-sectional; n = 3950; ExG = 3832	Male = 1896, female = 2054; N/I; N/I	Environmental; N/I; chronic; BTEXS	BTEXS, total xylenes (blood sample)	Mean diference: percentage change for total population (adjusted for benzene) = 1.8%

A: adults; BTEX: benzene, ethylbenzene, toluene, m-, p-xylenes, o-xylene; BTEXS = BTEX + styrene; CET: continuously exposed to toluene; CG: control group; OA: older adults; ExG: exposed group; IET: intermittently exposed to toluene; N/I: not informed; OR: odds ratio.

## DISCUSSION

In the literature, toluene exposure has been associated with hematological effects in humans, including reduced red blood cell count, hemoglobin concentration, increased white blood cell count, decreased and increased PC, and ITP.^9,11,14,22^

No conclusions could be drawn about the effects of toluene on PC based on the included studies, with some finding increases, decreases, or no effect. However, some confounders should be pointed out. The increased PC reported in some studies may be a consequence of a particular type of anemia, such as iron deficiency anemia, a recognized cause of reactive thrombocytosis, which may be due to increased megakaryopoiesis stimulated by such deficiency.^27^ Additionally, PCs were higher among smokers. One possible explanation for this may be that 1 or more chemical constituents of cigarette smoke stimulate bone marrow to increase production of certain blood components, such as white cells and platelets. The fact that young male smokers have higher white blood cell and PCs and lower hematocrit levels than nonsmokers is consistent with the hypothesis that inhalation of cigarette smoke causes inflammatory reactions, although studies have found that adult smokers have elevated hematocrit levels.^28^

It has been reported that low-level toluene exposure can lead to transient platelet agglutination, which can result in pseudo-thrombocytopenia. Therefore, in the included studies, the PC reduction in workers with long-term continuous toluene exposure could have been due to transient hyper-agglutination, a platelet synthesis disturbance, or increased platelet damage.^18,29^

As previously mentioned, 1 case report^10^ described exacerbated ITP after toluene exposure without associated substances. This suggests that toluene's relationship with ITP may be aggravation, rather than causation. A different case report^26^ described 2 cases of ITP after exposure to toluene diisocyanate. Hence, this specific isoform of toluene may play a role in the development of ITP.

There has been some debate about toluene's hematological effects, mainly due to cross-contamination with other volatile organic compounds, such as BTEX, being benzene a well-established hematotoxic substance.^9,30^ BTEX chemicals, which are often analyzed together, are thought to share some similar, non-carcinogenic effects.^14^ As pointed out in the literature, cross-contamination, especially with benzene, is a potential confounder for the overall results of our study^9,24,30^

Although exposure to high concentrations of BTEX, defined as > 1-20 ppm each^31,32^ is expected to cause neurotoxicity, it actually decreases the chance of hematotoxicity or carcinogenicity, as well as blood levels of benzene metabolites due to interaction between the compounds.^29,33,34,35,36^ Some studies have reported that the interaction between benzene and toluene decreases the hematotoxicity of benzene,^37,38^ while another reported that a mixture of toluene and benzene considerably increased the adverse effects of benzene on some hematological components, such as lymphocytes.^39^ These effects may be related to varying concentrations of benzene and toluene.^21,40^ Some studies on toxicokinetics and metabolism have reported that toluene reduces the toxicity of benzene, thus providing a protective effect.^40,42,43^

Most previous studies on the consequences of oil spills have compared an exposed group to an unexposed control group.^25^ Such studies can help clarify the acute effects of BTEX exposure, since the exposure period can be easily quantified, along with the exact agents. This is in contrast to environmental studies, which include broad populations and provide data on chronically exposed individuals with varying exposure periods.

Most exposed participants were environmentally exposed women, whose exposure was determined through biomarkers in blood samples. This contrasts with the common-sense expectation of men^44^ with industrial jobs involving toluene in the production process. The predominance of environmentally exposed participants was due to a small number of studies,^14,22^ which heavily skewed the data in that direction. However, we could not specifically account for the higher number of exposed women.

Nevertheless, we were able to shed some light on this understudied theme, collecting relatively comprehensive data that indicate contamination by a mixture of substances. Such cross-contamination could impair assessment of toluene's hematotoxicity in humans.

We must also point out certain study limitations, the first of which is the review design. We tried to mitigate the bias associated with narrative reviews by using a transparent search strategy that included: precise and uniform search terms (MeSH and DeCS) and inclusion and exclusion criteria; a search of several databases, including grey literature; and a manual search of the references of relevant articles to expand the search. Second, due to cross-contamination by other substances, mainly benzene, we could not determine the extent to which PC was affected by toluene in most of the studies. Furthermore, we may have disregarded other unmeasured confounders in the studies.

## CONCLUSIONS

Our findings highlight the scant data on toluene exposure and PC changes in the literature, especially exposure to toluene apart from other associated substances. Since the available data are contradictory, no clear conclusions can be drawn. The potential deleterious effects of toluene exposure on PC should be considered. Further research is needed to investigate this relationship, especially studies that include toluene exposure in isolation and the development or exacerbation of ITP through toluene diisocyanate.

## References

[1] National Library of Medicine, National Center for Biotechnology Information Toluene [Internet].

[2] National Library of Medicine, National Center for Biotechnology Information Benzene [Internet].

[3] National Library of Medicine, National Center for Biotechnology Information Ethylbenzene [Internet].

[4] U.S. Department of Health and Human Services, Public Health Service, Agency for Toxic Substances & Disease Registry CATSDR) (2017). Toxicological profile for toluene.

[5] Foster LMK, Tannhauser M, Tannhauser SL (1994). Toxicologia do tolueno: aspectos relacionados ao abuso. Rev Saude Publica.

[6] Camara-Lemarroy CR, Rodríguez-Gutiérrez R, Monreal-Robles R, González-González JG (2015). Acute toluene intoxication-clinical presentation, management and prognosis:
a prospective observational study. BMC Emerg Med.

[7] Agency for Toxic Substances & Disease Registry (ATSDR) (2014). Medical management guidelines for toluene.

[8] Cruz SL, Rivera-Garcia MT, Woodward JJ (2014). Review of toluene action: clinical evidence, animal studies and molecular
targets. J Drug Alcohol Res.

[9] Chen Q, Sun H, Zhang J, Xu J, Ding Z (2019). The hematologic effects of BTEX exposure among elderly residents in
Nanjing: a cross-sectional study. Environ Sci Pollut Res.

[10] Dodson VN (1966). Purpura and chemicals. J Occup Med.

[11] Jennings GH, Gower ND (1963). Thrombocytopenic purpura in toluene di-isocyanate workers. Lancet.

[12] Doherty BT, Kwok RK, Curry MD, Ekenga C, Chambers D, Sandler DP (2017). Associations between blood BTEXS concentrations and hematologic parameters
among adult residents of the U.S. Gulf States. Environ Res.

[13] Pelallo-Martínez NA, Batres-Esquivel L, Carrizales-Yáñez L, Díaz-Barriga FM (2014). Genotoxic and hematological effects in children exposed to a chemical
mixture in a petrochemical area in Mexico. Arch Environ Contam Toxicol.

[14] Watson CV, Naik S, Lewin M, Ragin-Wilson A, Irvin-Barnwell E (2021). Associations between select blood VOCs and hematological measures in NHANES
2005-2010. J Expo Sci Environ Epidemiol.

[15] Qu Q, Shore R, Li G, Jin X, Chen LC, Cohen B (2002). Hematological changes among Chinese workers with a broad range of benzene
exposures. Am J Ind Med.

[16] Brasil, Presidência da República, Casal Civil, Subchefia para Assuntos Jurídicos (2003). Lei nº 10.741, de 1º de outubro de 2003.

[17] Brasil, Presidência da República, Casal Civil, Subchefia para Assuntos Jurídicos (1990). Lei nº 8.069, de 13 de julho de 1990.

[18] Shih HT, Yu CL, Wu MT, Liu CS, Tsai CH, Hung DZ (2011). Subclinical abnormalities in workers with continuous low-level toluene
exposure. Toxicol Ind Health.

[19] Haro-García L, Vélez-Zamora N, Aguilar-Madrid G, Guerrero-Rivera S, Sánchez-Escalante V, Muñoz SR (2012). Alteraciones hematológicas en trabajadores expuestos
ocupacionalmente a mezcla de benceno-tolueno-xileno (BTX) en una fábrica de
pinturas. Rev Peru Med Exp Salud Publica.

[20] Ezejiofor TIN, Ezejiofor AN, Iwuala MOE (2016). Haematological indicators of exposure to petroleum products in petroleum
refining and distribution industry workers in Nigeria. J Clin Toxicol.

[21] Samadi MT, Shakerkhatibi M, Poorolajal J, Rahmani A, Rafieemehr H, Hesam M (2019). Association of long term exposure to outdoor volatile organic compounds
(BTXS) with pro-inflammatory biomarkers and hematologic parameters in urban adults: A
cross-sectional study in Tabriz, Iran. Ecotoxicol Environ Saf.

[22] Cakmak S, Cole C, Hebbern C, Andrade J, Dales R (2020). Associations between blood volatile organic compounds, and changes in
hematologic and biochemical profiles, in a population-based study. Environ Int.

[23] Park MY, Kim DH, Park HJ (2012). Delayed onset of parkinsonism after industrial toxic gases
intoxication. Mov Disord.

[24] Tharr D, Kudla I (1997). Case studies exposure to benzene-contaminated toluene and bone marrow
disorders—a retrospective exposure assessment. Appl Occup Environ Hyg.

[25] Choi YH, Hong JY, Lee MS (2017). A retrospective mid- and long-term follow-up study on the changes in
hematologic parameters in the highly exposed residents of the Hebei Spirit oil spill in
Taean, South Korea. Osong Public Health Res Perspect.

[26] Eze A, Eluke BC, Eluke CC, Ezigbo E, Uzoma I (2019). The effect of chronic occupational exposure to petroleum products on
haematological and biochemical parameters of petrol attendants. J Adv Med Med Res.

[27] Kadikoylu G, Yavasoglu I, Bolaman Z, Senturk T (2006). Platelet parameters in women with iron deficiency anemia. J Natl Med Assoc.

[28] Tell GS, Grimm Jr RH, Vellar OD, Theodorsen L (1985). The relationship of White cell count, platelet count, and hematocrit to
cigarette smoking in adolescents: the Oslo Youth Study. Circulation.

[29] Aakhus AM, Smith-Kielland A, Ripel A, Solum NO (1991). Effects of toluene on platelet membrane glycoprotein lb and actin-binding
protein. Biochem Pharmacol.

[30] Yin S, Li G, Hu Y, Zhang X, Jin C, Inoue O (1987). Symptoms and signs of workers exposed to benzene, toluene or the
combination. Ind Health.

[31] U.S. Department of Health and Human Services, Public Health Service, Agency for Toxic Substances & Disease Registry CATSDR) (2004). Interaction profile for: benzene, toluene, ethylbenzene, and xylenes
(BTEX).

[32] Brasil, Ministério do Trabalho e Emprego, Secretaria de Segurança e Saúde no Trabalho (1995). Portaria n° 14, de 20 de dezembro de 1995. NR 15 - Atividades e
operações insalubres. Anexo n° 13-A.

[33] Haddad S, Tardif R, Charest-Tardif G, Krishnan K (1999). Physiological modeling of the toxicokinetíc interactions in a
quaternary mixture of aromatic hydrocarbons. Toxicol Appl Pharmacol.

[34] Haddad S, Tardif R, Viau C, Krishnan K (1999). A modeling approach to account for toxicokinetic interactions in the
calculation of biological hazard index for chemical mixtures. Toxicol Lett.

[35] Haddad S, Béliveau M, Tardif R, Krishnan K (2001). A PBPK modeling-based approach to account for interactions in the health
risk assessment of chemical mixtures. Toxicol Sci.

[36] Tardif R, Charest-Tardif G, Brodeur J, Krishnan K (1997). Physiologically based pharmacokinetic modeling of a ternary mixture of
alkyl benzenes in rats and humans. Toxicol Appl Pharmacol.

[37] Ikeda M, Otsuji H, Imamura T (1972). In vivo suppression of benzene and styrene oxidation by co-administered
toluene in rats and effects of phenobarbital. Xenobiotíca.

[38] Tunek A, Olofsson T, Berlin M (1981). Toxic effects of benzene and benzene metabolites on granulopoietic stem
cells and bone marrow cellularity in mice. Toxicol Appl Pharmacol.

[39] Bird MG, Wetmore BA, Letinski DJ, Nicolich M, Chen M, Schnatter AR (2010). Influence of toluene co-exposure on the metabolism and genotoxicity of
benzene in mice using continuous and intermittent exposures. Chem Biol Interact.

[40] Sato A, Nakajíma T (1979). Dose-dependent metabolic interaction between benzene and toluene in vivo
and in vitro. Toxicol Appl Pharmacol.

[41] Andrews LS, Lee EW, Witmer CM, Kocsis JJ, Snyder R (1977). Effects of toluene on the metabolism, disposition and hemopoietic toxicity
of [3H]benzene. Biochem Pharmacol.

[42] Gradiski D, Bonnet P, Duprat P, Zissu D, Magadur JL, Guenier JP (1981). Etude toxicologique, chronique par inhalation chez le rat de l'association
benzène-toluène. Toxicol Eur Res.

[43] Plappert U, Barthel E, Seidel HJ (1994). Reduction of benzene toxicity by toluene. Environ Mol Mutagen.

[44] Battaus MR, Monteiro Ml (2013). Perfil sociodemográfico e estilo de vida de trabalhadores de uma
indústria metalúrgica. Rev Bras Enferm.

